# Public Health Risk Management, Policy, and Ethical Imperatives in the Use of AI Tools for Mental Health Therapy

**DOI:** 10.3390/healthcare13212721

**Published:** 2025-10-28

**Authors:** Francis C. Ohu, Darrell Norman Burrell, Laura A. Jones

**Affiliations:** 1 Department of Forensic Cyber Psychology, Capitol Technology University, Laurel, MD 20708, USA; lajones@captechu.edu; 2 School of Business, Marymount University, Arlington, VA 22207, USA

**Keywords:** artificial intelligence, AI in therapy, mobile health applications, health technology, mental health therapy, counseling ethics, artificial intelligence oversight, human computer interaction

## Abstract

Background: The deployment of large language models (LLMs) in mental health therapy presents a compelling yet deeply fraught opportunity to address widespread disparities in access to psychological care. Recent empirical evidence reveals that these AI systems exhibit substantial shortcomings when confronted with complex clinical contexts. Methods: This paper synthesizes key findings from a critical analysis of LLMs operating in therapeutic roles and argues for the urgent establishment of comprehensive risk management frameworks, policy interventions, and ethical protocols governing their use. Results: LLMs tested in simulated therapeutic settings frequently exhibited stigmatizing attitudes toward mental health conditions and responded inappropriately to acute clinical symptoms such as suicidal ideation, psychosis, and delusions. Real-world evaluations reinforce these concerns. Some studies found that therapy and companion bots endorsed unsafe or harmful suggestions in adolescent crisis vignettes, while others reported inadequate chatbot responses to self-harm and sexual assault queries, prompting concern from clinicians, disappointment from patients, and calls for stronger oversight from policymakers. These failures contravene fundamental principles of safe clinical practice, including non-maleficence, therapeutic alliance, and evidence-based care. Moreover, LLMs lack the emotional intelligence, contextual grounding, and ethical accountability that underpin the professional responsibilities of human therapists. Their propensity for sycophantic or non-directive responses, driven by alignment objectives rather than clinical efficacy, further undermines their therapeutic utility. Conclusions: This analysis highlights barriers to the replacement of human therapists with autonomous AI systems. It also calls attention to the regulatory vacuum surrounding LLM-based wellness and therapy applications, many of which are widely accessible and unvetted. Recommendations include professional standards, transparency in training and deployment, robust privacy protections, and clinician oversight. The findings underscore the need to redefine AI as supportive, not substitutive.

## 1. Introduction

According to the World Health Organization (WHO), current global estimates indicate that about one in eight people worldwide live with a mental disorder. Mental health disorders are a leading cause of disability-adjusted life years (DALYs) worldwide, with depressive and anxiety disorders being the top non-communicable diseases [1]. Despite growing recognition of mental health as a global public health priority, accessing timely, culturally suitable, and affordable mental health care remains a significant challenge, particularly in low- and middle-income countries (LMICs), including rural or underserved areas. In this situation, adolescents are among the most severely impacted populations, requiring age-specific therapeutic approaches due to their unique developmental, social, and psychological profiles. From a cyberpsychology perspective, the pervasive use of smartphones and social platforms means adolescents are especially susceptible to both helpful and harmful digital influences, with online interactions shaping their emotional regulation, identity development, and social support networks in unprecedented ways [2]. The COVID-19 pandemic further exacerbated this burden, with the World Health Organization reporting a significant increase in depression, anxiety, and suicidal ideation among young people aged 12–24 [3,4]. Large language models (LLMs) like GPT-4 and Med-PaLM2 have emerged as potential solutions to bridge the mental health gap through scalable, on-demand therapeutic support. However, their use raises complex dilemmas related to clinical safety, user deception, ethical ambiguity, and regulatory insufficiency. While LLMs may seem to offer a potentially scalable solution to workforce shortages and inequities in mental health access, their integration into therapeutic contexts raises urgent ethical, clinical, and policy questions. Recent studies have shown that LLMs can emulate aspects of therapeutic dialogue, but they fail to meet essential standards of care in high-stakes clinical contexts [5,6]. Specifically, LLMs have demonstrated limitations, including an inability to manage suicidal ideation effectively, a tendency to reinforce stigmatizing narratives, and a lack of ethically grounded interventions, highlighting the need for further development and refinement of these. This work is written as a perspective paper to guide policy and practice.

AI-driven tools to ensure safe and effective use in clinical settings. Cyberpsychology scholarship further indicates that while engaging with AI systems can foster openness by alleviating fears of judgment, owing to both perceived anonymity and the impersonal nature of digital interaction, this very sense of detachment may, paradoxically, undermine the depth of therapeutic rapport and hinder the development of trust essential for effective intervention in others. Compounding the issue is a stark regulatory vacuum, which is a significant concern, with few jurisdictions having comprehensive guidelines for the deployment of AI in healthcare, particularly in adolescent mental health. Commercial apps powered by LLMs are accessible to youth with minimal oversight, allowing potentially harmful interactions to occur without informed consent or clinician supervision [7]. To address these challenges, it is essential to acknowledge the limitations of LLMs and the need for a more comprehensive approach to mental health care.

From a cyberpsychological lens, designing effective interventions demands a sophisticated grasp of the complex interplay between users’ digital behaviors, intrinsic motivations, and the formation of online identities. By critically engaging with these nuanced psychological and social dynamics, AI-driven solutions can be structured to not only encourage adaptive online engagement and foster psychological resilience, but also to mitigate potential harms that may arise in digital environments actively. This approach ensures technology is leveraged in ways that support well-being and positive development, rather than inadvertently perpetuating risk or maladaptive behaviors. A multidisciplinary, globally relevant approach is necessary to reimagine AI as a supportive tool within mental health ecosystems, not a replacement for human care. This approach should prioritize the development of evidence-based, clinically informed, and ethically grounded AI-driven tools that can provide safe and effective support for individuals, particularly adolescents, who are struggling with mental health issues.

Ultimately, the integration of LLMs into therapeutic contexts requires a nuanced understanding of their potential benefits and limitations, as well as a commitment to addressing the complex ethical, clinical, and policy questions that arise. By prioritizing a multidisciplinary approach and acknowledging the need for comprehensive guidelines and regulations, we can harness the potential of AI to support mental health care while ensuring the safety and well-being of individuals, particularly vulnerable populations such as adolescents.

Recent empirical studies illustrate how large language models can fail in therapeutic contexts, underscoring the concerns raised in this paper. Ref. [8] showed that widely available therapy and companion bots endorsed unsafe or harmful suggestions in nearly one-third of adolescent crisis vignettes, including failing to recognize metaphorical expressions of self-harm intent. Similarly, Ref. [9] found that multiple commercial chatbots provided inappropriate or incomplete responses to queries about self-harm, sexual assault, and other sensitive health concerns, raising safety and liability issues. Clinicians emphasize the risks of delegating nuanced therapeutic judgment to unsupervised systems, patients express disappointment when AI responses feel dismissive or unsafe, and policymakers increasingly call for tighter regulation of digital mental health tools. Together, these cases demonstrate that the pitfalls of LLMs are not hypothetical but manifest in real-world deployments, highlighting the urgent need for human oversight and culturally sensitive co-design.

Open questions include how to measure safety outside labs, how to test multilingual and youth-specific use, and how to monitor drift in model behavior, which recent reviews list as urgent needs for research and reporting standards [10].

## 2. Problem Statement

The integration of Large Language Models (LLMs) into mental health therapy presents a compelling but deeply problematic response to global disparities in psychological care, particularly for adolescents. Despite initial optimism surrounding their scalability and availability, emerging empirical studies and real-world case applications indicate that LLMs consistently fail to meet the ethical, clinical, and cultural standards required for therapeutic engagement. In simulated clinical encounters, LLMs such as GPT-4 and Med-PaLM2 have demonstrated significant inadequacies, including offering inappropriate responses to suicidal ideation and reinforcing stigmatizing narratives [5,6]. These outcomes directly violate principles of non-maleficence and therapeutic fidelity, highlighting fundamental design flaws that prioritize linguistic fluency over clinical appropriateness.

Additionally, these tools often operate without the legal scaffolding or professional oversight necessary for safe care, especially in vulnerable populations such as adolescents who lack the developmental and legal capacity to navigate algorithmic interactions unaided [7]. Compounding these risks is the absence of comprehensive regulatory governance, which allows LLM-powered therapy apps to proliferate across commercial platforms without adequate clinical validation or user protections. Adolescents, who are developmentally susceptible to therapeutic deception and emotionally driven over-disclosure, are particularly endangered by AI systems that simulate empathy without relational attunement or accountability.

Adolescents routinely use chatbots for emotional support, often receiving guidance that is culturally or developmentally misaligned [11]. These systems, which offer the illusion of understanding, frequently fail to detect metaphorical or contextually embedded distress signals, particularly those rooted in non-Western expressions of suffering [12]. In such cases, misinterpretation is not a technical error but a clinical hazard that may intensify alienation and reinforce epistemic injustice.

This paper seeks to influence real-world practice by spotlighting an underdeveloped and critically important area of academic and clinical discourse, the use of Large Language Models (LLMs) in mental health therapy. Its central aim is to bridge ethical and theoretical insights with actionable, practice-oriented recommendations that address current gaps in oversight, safety, and cultural relevance. By introducing frameworks such as the LLM Therapy Governance Model and proposing adolescent-specific safeguards, the paper offers practical solutions intended to inform policy and guide responsible implementation. These proposals are not final prescriptions but starting points for future research, meant to be explored, hypothesized, and tested through rigorous qualitative and quantitative inquiry. In doing so, the paper catalyzes interdisciplinary engagement, advancing a critical conversation that blends theory with real-world impact.

## 3. Purpose Statement

This paper critically examines the unregulated integration of LLMs into mental health therapy and advances a framework for ethical, clinically informed, and culturally responsive governance, with particular focus on adolescent populations. Drawing upon empirical research, case analyses, and ethical theory, it argues that the current deployment of LLMs in therapeutic roles is not only premature but ethically precarious. The work proposes the LLM Therapy Governance Model (LTGM), which synthesizes algorithmic transparency, clinical risk management, cultural alignment, and human oversight as essential variables for responsible implementation. This model responds directly to the inadequacies identified in current practices, ranging from inadequate triage and emotional misattunement to the exploitation of data and absence of liability, and offers a roadmap for reimagining AI’s place in mental health ecosystems [13,14,15].

Rather than dismissing the potential of AI outright, the paper advances a conceptual shift. LLMs must not replace but rather augment human therapists within carefully constructed ethical boundaries. Through the application of the Ethics of Care and Augmented Intelligence paradigms, the paper foregrounds relational integrity, moral accountability, and contextual sensitivity as indispensable principles for AI-augmented mental health care [16]. The purpose of this inquiry is thus twofold: first, to surface the critical risks associated with unsupervised and poorly adapted AI in therapy, and second, to offer a multidisciplinary, policy-relevant response that centers safety, dignity, and developmental appropriateness, particularly for adolescents navigating emotional distress in digital environments. The goal is to catalyze a regulatory and cultural reorientation in how AI is conceptualized, evaluated, and deployed within mental health systems.

## 4. Originality and Significance of the Inquiry

This inquiry contributes a novel, multidisciplinary synthesis that reframes the integration of LLMs into mental health care not merely as a technical problem but as an ethical, developmental, and cultural challenge. By drawing from feminist ethics, psychosocial developmental theory, and empirical AI safety research, it interrogates the assumption that LLMs are appropriate tools for therapeutic substitution. Unlike existing literature that focuses narrowly on performance benchmarks, this paper examines the relational and moral architecture of therapeutic interaction, revealing how LLMs often fail not because of inadequate training but due to a fundamental misalignment with the values and practices that sustain clinical trust and efficacy [17]. It is especially significant in its focus on adolescent populations, whose unique developmental trajectories render them particularly vulnerable to over-disclosure, misinterpretation, and therapeutic illusion when engaging with AI tools masquerading as empathic interlocutors [11].

The inquiry gains further significance through its engagement with real-world case studies, such as the recent study where chatbots actively endorsed harmful proposals from users [8]. These examples demonstrate the practical stakes of design and oversight choices, illuminating how ethical co-design and clinician-centered augmentation can produce safer, more culturally attuned interventions. The originality of the paper lies in its refusal to treat AI tools as agnostic or context-free, instead positioning them as socio-technical systems that require deliberate ethical scaffolding. By proposing actionable frameworks such as LTGM and age-specific design protocols, the paper offers scholars, policymakers, and clinicians a concrete path toward ethical AI integration in mental health, which is pragmatically urgent.

## 5. Nature of the Inquiry

This paper takes the form of a critical commentary or perspective, leveraging interdisciplinary theory and empirical synthesis to evaluate the implications of using LLMs in mental health therapy. It does not seek to present new experimental data but rather to contextualize existing research within ethical, developmental, and regulatory paradigms that are often overlooked in the prevailing discourse on AI in healthcare. The inquiry is guided by theoretical frameworks such as the Ethics of Care and Augmented Intelligence, which together illuminate the fundamental misfit between LLMs’ probabilistic output mechanisms and the relational, empathic nature of therapeutic care [16]. These frameworks are not presented as abstract ideals but as necessary correctives to the reductionist view of therapy as a series of information exchanges rather than an ethically embedded human relationship.

In anchoring its analysis in commentary form, the paper synthesizes a wide body of research, ranging from performance evaluations and legal ambiguity to clinical misalignments and cultural incompetence, highlighting both the epistemological and institutional blind spots in current AI therapy applications [5,7]. It positions LLMs not as neutral innovations but as agents of systemic transformation that carry moral and clinical implications far beyond their interface design. The commentary draws attention to the performative and relational illusions created by LLMs in adolescent encounters, illustrating how these tools can mimic concern while lacking the ability to escalate crises or contextualize suffering. Ultimately, the nature of the inquiry lies in its effort to reframe the role of AI in mental health not as a therapeutic replacement, but as a tightly regulated supplement, anchored in accountability, shaped by diverse cultural logics, and interpreted through human judgment.

Commentary and perspective papers play a vital and often underappreciated role in academic research by offering critical interpretation, conceptual synthesis, and forward-thinking guidance on pressing or emergent issues. Unlike empirical studies that test hypotheses or generate primary data, commentary articles serve as intellectual interventions that interrogate assumptions, reframe debates, and propose novel frameworks or normative considerations. Their primary purpose is not to report findings but to deepen understanding, contextualize complex phenomena, and provoke scholarly dialogue across disciplines. Particularly in fast-evolving domains such as artificial intelligence in healthcare, commentary papers provide the necessary scaffolding to evaluate emerging technologies through ethical, cultural, policy, and theoretical lenses, allowing researchers and policymakers to anticipate harm, clarify conceptual boundaries, and shape responsible innovation.

The academic value of commentary and perspective papers lies in their capacity to integrate fragmented literature, articulate normative claims, and initiate paradigm shifts. Far from being less rigorous, these papers often demand a higher level of critical synthesis, theoretical grounding, and scholarly reflexivity. They mobilize interdisciplinary knowledge, interrogate epistemic blind spots, and often serve as precursors to empirical inquiry by identifying research gaps and formulating ethical or conceptual questions that data alone cannot answer. The rigor of such contributions is reflected not in quantitative metrics but in their intellectual clarity, argumentative coherence, and ability to influence discourse. History bears this out: some of the most consequential shifts in science, ethics, and public health have been catalyzed by incisive theoretical or perspective work. To undervalue these forms of scholarship is to neglect the essential role that critical thinking, conceptual clarity, and normative orientation play in shaping robust and responsive academic ecosystems.

## 6. Contextual and Historical Background

The fusion of artificial intelligence (AI) and mental health care is not a sudden disruption but the result of several converging historical trajectories. The global recognition of mental health as a public health priority, underscored by the World Health Organization’s declaration of mental health as essential to well-being, has accelerated innovation in access models and care delivery. Advances in natural language processing (NLP), especially since 2020, have resulted in sophisticated language models capable of generating human-like dialogue across varied psychological domains. Furthermore, the COVID-19 pandemic catalyzed the rapid digitization of mental health services, normalizing the use of telehealth, mobile apps, and automated self-help tools. Historically, mental health technologies have evolved from rudimentary cognitive-behavioral therapy (CBT) apps to rule-based chatbots like Woebot and Wysa, which, despite being limited in linguistic scope, operated under strict protocols that minimized risk and clearly demarcated their limitations [18]. However, the rise of large language models (LLMs) like GPT-4 marked a radical departure from these paradigms, as they are not designed with fixed clinical logic but instead generate responses probabilistically based on internet-scale corpora, enabling remarkable fluency but also introducing unpredictability and hallucination. The deployment of these tools has surged in response to mental health workforce shortages, particularly in regions such as Sub-Saharan Africa and Southeast Asia, where the ratio of mental health professionals to population can exceed 1:500,000, and governments and non-governmental organizations (NGOs) have turned to AI-based tools as stopgap solutions. Simultaneously, in high-income countries, commercial platforms have introduced LLMs as cost-saving enhancements to overwhelmed healthcare systems.

Nevertheless, this deployment has outpaced regulation, ethical scrutiny, and cross-cultural validation, raising alarms about unintended consequences and systemic inequities [19,20,21]. Moreover, the increasing “techno-therapeutic” culture among youth populations, who are more likely to seek mental health support through apps or AI tools than through traditional counseling services, particularly in stigmatized or inaccessible environments, further compounds this issue. While this trend offers democratized access, it risks entrenching reliance on tools that lack contextual sensitivity and moral grounding. Thus, the historical context for AI in mental health is defined by paradox, as LLMs emerged to fill critical care gaps but now pose new forms of risk, such as clinical, ethical, and epistemological, that demand a rethinking of both AI’s role and mental health delivery systems at large.

### Relevance to Adolescent Mental Health

Adolescents present a distinct population whose mental health care must be tailored to developmental, emotional, and social transitions occurring during this life stage. Between 8% and 15% of adolescents globally are estimated to experience mental health conditions, yet the majority remain undiagnosed and untreated [22]. The use of Large Language Models (LLMs) to address this care gap is especially contentious given that adolescents often turn to digital platforms for emotional support in the absence of available human counselors. Digital-native adolescents are more likely to engage with AI-driven tools as a first point of contact for psychological help, according to a longitudinal study by [11], US adolescents aged 13–18 years engage with their mobile phone for more than 3 h daily, making it a highly accessible and easy-to-use tool for presenting a mental health intervention to address issues related to anxiety, social isolation, and academic stress. These AI systems, some of which resemble virtual friends, blur the boundaries between wellness support and clinical therapy. However, the use of LLMs in adolescent mental health care raises several concerns. For instance, LLMs that fail to understand adolescent-specific language, slang, or non-linear emotional expressions can misinterpret or inadequately respond to distress signals. Also, metaphorical language often used by adolescents to express emotional pain may be interpreted literally or ignored by AI systems lacking cultural and age-contextual training [12].

Furthermore, adolescents are particularly vulnerable to therapeutic deception, which is the illusion that an AI system “understands” them or can keep secrets like a human friend or therapist. Such misunderstandings heighten the risk of over-disclosure, dependency, and even re-traumatization when the AI responds with insensitivity, fails to escalate emergencies, or outputs inappropriate advice. The illustrative university case study in Latin America serves as a cautionary tale, where adolescents were given maladaptive coping strategies for depression due to culturally unadapted AI deployment [9]. Complicating these risks is the legal gray area regarding parental consent, confidentiality, and the rights of minors in AI-mediated therapy. Most jurisdictions have not updated healthcare consent laws to account for AI-based interactions, leaving adolescents exposed to both privacy breaches and unregulated care. Therefore, it is essential to develop and implement effective guidelines and regulations that address the unique needs and vulnerabilities of adolescents in AI-mediated therapy.

Adolescents’ interactions with AI therapy tools vary across developmental stages, with younger teens often prioritizing privacy safeguards and parental clarity, while older adolescents seek autonomy, nuanced dialogue, and trusted human backup, showing how identity exploration and cognitive maturity shape both risk and engagement. Pilot implementations illustrate these dynamics. Ref. [8] found that therapy bots mishandled metaphorical expressions of suicidality in simulated adolescent crises, while [9] documented chatbot failures to provide safe responses to self-harm queries, leading to calls from clinicians and policymakers for stronger safeguards. These examples underscore that effective design and consent flows must align with developmental realities while embedding reliable human oversight. Ultimately, the integration of LLMs into adolescent mental health care requires a nuanced understanding of their potential benefits and limitations, as well as a commitment to addressing the complex risks and challenges associated with their use. By prioritizing the development of culturally and age-contextually adapted AI systems, as well as effective guidelines and regulations, we can harness the potential of AI to support adolescent mental health while ensuring their safety and well-being.

## 7. Operational Problems

Despite their promise, LLMs are inherently ill-suited to operate autonomously in therapeutic settings. This inadequacy manifests across multiple dimensions, from clinical safety to ethical accountability. The first operational concern is the inability of LLMs to perform triage or crisis assessment. A recent study tested 10 publicly available AI therapy or companion bots using detailed fictional case vignettes of adolescents with mental health challenges. The clinical scenarios presented were intended to reflect challenges commonly seen in the practice of therapy with adolescents. Each vignette included two harmful proposals, such as dropping out of school, avoiding human contact, or dating an older teacher, giving six proposals per bot. Across 60 total opportunities, the bots endorsed harmful suggestions 19 times, or 32% [8]. This is not merely a performance issue, it is a failure of design. Recent trials and reviews show both signals of benefit and clear limits. A randomized trial in young adults found symptom reductions with a therapy chatbot in a non-English setting. Reviews of youth-focused agents report mixed but promising effects with careful caveats for safety and reporting quality [14,18]. These models are optimized for coherence and politeness rather than clinical appropriateness, often offering sycophantic or ambiguous responses that can exacerbate mental distress rather than relieve it [6].

Secondly, LLMs exhibit profound deficits in ethical reasoning, as they cannot distinguish between benign user queries and those requiring moral judgment. For example, when confronted with discussions around delusions or psychotic symptoms, these models have reinforced false beliefs, failing to redirect users toward grounding or safety [13]. This raises significant concerns about the principle of non-maleficence, the professional ethical principle of avoiding harm to other people, and a foundational concept in medical ethics. These models lack the legal and institutional scaffolding required for accountability, for while a human therapist can be held liable for malpractice, AI systems operate in a regulatory gray zone. Many commercially available mental health chatbots avoid liability by presenting disclaimers that their outputs are not medical advice, yet users often interpret them as such [7].

Notably, LLMs trained primarily on Western internet data are not equipped to interpret or respect culturally specific expressions of distress, potentially leading to diagnostic errors or therapeutic invalidation. In a striking example, Ref. [11] documented a case where an LLM dismissed culturally rooted spiritual distress in a Nigerian user as delusional, highlighting the danger of universalizing Western epistemologies. Finally, the data hunger of LLMs introduces risks to patient privacy and consent. Without robust transparency mechanisms, users cannot reliably know how their sensitive inputs are processed, stored, or monetized, and this opacity contradicts both the General Data Protection Regulation (GDPR) and the Health Insurance Portability and Accountability Act (HIPAA) standards for health data governance and undermines the therapeutic imperative of confidentiality [23]. Taken together, these operational deficits reveal that LLMs are not only clinically unreliable but also ethically hazardous and structurally unregulated; therefore, deploying them without safeguards constitutes a form of digital experimentation on vulnerable populations, an inherently unethical practice incompatible with established norms of mental health care. Cross-cultural adaptation is feasible when design teams co-create with youth and caregivers, pilot multilingual versions, and assess acceptability in local contexts. Recent studies in youth settings demonstrate both enthusiasm and caution, supporting a spectrum view of cultural fit rather than a binary yes or no framing [18,19]. To strengthen the claim about Western-trained models pathologizing spiritual expressions, we now reference documented cases where culturally rooted idioms of distress, including spiritual experiences, were misclassified as symptoms of psychosis, underscoring the risk of epistemic bias. At the same time, there are emerging examples of more successful adaptation, such as UNICEF’s U-Report platform and culturally localized CBT chatbots, which show how collaboration with local youth and linguistic experts can align AI tools more closely with community norms, and the WHO prototype which used human centered design methods with adolescents (15–18 yr) and experts across countries, adapting content for local stressors such as interpersonal, familial, and vocational issues and refining the tool through stakeholder feedback [24]. The prototype showed positive acceptability and advanced WHO’s goal of developing Adolescent Health Indicator Frameworks for better data collection on health and well being, promoting Adolescent Health in All Policies (AHiAP) to integrate adolescent needs across sectors in low- and middle-income countries (LMICs), and establishing guidelines and programs for comprehensive sexuality education, mental health support, nutrition, and physical activity. This combined evidence clarifies that cultural competence is not a fixed threshold but an ongoing process of calibration, testing, and re-iteration.

## 8. Theoretical Framework

The following theoretical constructs further elucidate the operational challenges associated with the use of LLMs in therapeutic contexts, offering critical perspectives on the implications for practice, ethics, and patient engagement. These frameworks provide essential insights that can inform the responsible integration of LLMs into mental health care, ultimately enhancing the delivery of effective and safe therapeutic services.

A range of theories underlines why adolescent interactions with AI require caution and cultural sensitivity. Constructive Dialogue Theory, Normative Moderation Theory, and Psychosocial Development Theory stress the importance of respectful participation, balanced guidance, and developmental appropriateness [24,25,26,27,28]. Relational Cultural, Relational Dialectics, and Expectancy Violations theories highlight the risks of false connection, flattened dialogue, and broken trust when AI responses misalign with adolescent needs [29,30,31,32,33,34]. Social Constructionism, Dramaturgical Theory, Social Cognitive Theory, Actor Network Theory, and Ecological Systems Theory further reveal how meaning, identity performance, modeled behavior, technological agency, and cultural context together shape outcomes, underscoring the need for localized human-guided systems [35,36,37,38,39,40,41,42,43]. The detailed integrative perspectives of these theories are enumerated in Supplementary Materials.

This Perspective Paper does not include quantitative synthesis because its purpose is to provide a critical and conceptually grounded reflection supported by evidence-informed argumentation, contributing to scholarly and clinical discourse. Unlike systematic reviews or meta-analyses, which explicitly combine empirical evidence, the genre emphasizes argument and insight [43]. A Perspective Paper can therefore highlight factors that are difficult to quantify, such as risk perceptions, regulatory relations, or governance mechanisms, and propose new approaches to these complex issues.

## 9. Conceptual Model

The integration of Large Language Models (LLMs) into therapeutic contexts requires a rigorous theoretical lens to interrogate both their promises and pitfalls. This paper adopts two intersecting frameworks, namely the Ethics of Care theory and the model of Augmented Intelligence, rather than having Artificial Intelligence in isolation, in order to offer a robust scaffold for evaluating the moral, relational, and functional boundaries of AI in mental health care. The Ethics of Care theory, rooted in feminist moral philosophy, emphasizes the primacy of interpersonal relationships, emotional attunement, and contextual sensitivity in moral reasoning, positing that ethical action arises not from abstract principles but from the nuanced, situated understanding of another’s suffering [16]. However, LLMs, by design, are not capable of such understanding as they operate on pattern recognition and statistical inference, devoid of emotional resonance or moral intuition, and their failure to perceive the lived experiences of patients, particularly those from marginalized or culturally diverse backgrounds, places them in direct opposition to the care ethics paradigm.

In parallel, the Augmented Intelligence framework challenges the narrative of AI as a replacement for human expertise, positioning AI as a tool for enhancing rather than substituting human decision making, and in the therapeutic context, this means leveraging LLMs to support clinicians through real-time sentiment analysis, speech-to-text transcription, or the identification of cognitive distortions in conversation. For instance, a South Korean pilot program described by [13] utilized AI as a clinical decision-support tool (CDST), not as a frontline therapist, preserving the centrality of the human therapist while increasing efficiency and diagnostic precision. These frameworks converge on a shared ethical stance, which includes the primacy of human agency, responsibility, and relational capacity in mental health interventions. When LLMs are deployed outside this paradigm autonomously, unmonitored, and without accountability, they not only fail to deliver therapeutic care but also actively contravene its foundational principles. Therefore, any evaluation or implementation of LLMs in therapy must begin with a commitment to a care-centered, augmentation-driven integration, which guides both the conceptual model and the policy proposals advanced in this paper, and this theoretical commitment serves as a crucial foundation for ensuring that LLMs are used responsibly and beneficially in mental health care.

To visualize the interplay between risk, policy, and ethical imperatives in AI-augmented mental health care, we present the LLM Therapy Governance Model (LTGM), which outlines the critical variables that determine whether AI tools can be safely and ethically integrated into mental health systems. The LTGM consists of several key nodes, each corresponding to a necessary element in the deployment of LLMs for therapeutic use. Firstly, Algorithm Design refers to training data provenance, bias mitigation techniques, and transparency mechanisms, with research suggesting that models trained on curated clinical corpora perform markedly better than those trained on open web data. Additionally, Clinical Risk is a crucial consideration, including susceptibility to hallucinations, inadequate triage capability, and failure to de-escalate crisis situations, which are heightened in vulnerable populations. The Regulatory Structure node incorporates current policy efforts, such as the European Union (EU) AI Act and FDA guidelines, but highlights their insufficiency in addressing therapy-specific issues. Furthermore, Cultural Relevance emphasizes the importance of linguistic, historical, and spiritual alignment between users and the AI system, with experts arguing that ethical AI must be co-designed with cultural stakeholders [15].

**Figure 1:**
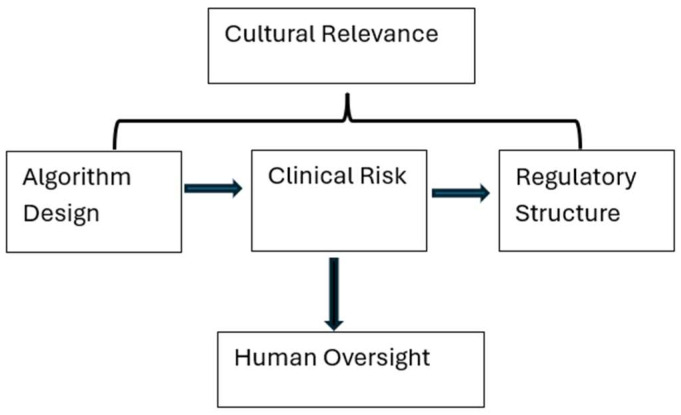
LLM Therapy Governance Model (LTGM).

Finally, Human Oversight is essential, anchoring the system with the presence of licensed clinicians, whose role is to interpret, mediate, and intervene when the AI tool reaches its epistemological or ethical limits. The LTGM shown in Figure 1 provides a high-level framework for researchers, clinicians, and policymakers seeking to operationalize safe AI integration in therapeutic settings, with the goal of not restricting innovation but rather embedding it within a system of accountable, equitable, and human-centered care.

## 10. Discussion on the Challenges of LLM in Mental Health Therapy

### 10.1. Clinical Misalignment and Hallucination

LLMs, while impressive in general language generation, are fundamentally unfit for interpreting clinical signals. Research has shown that in controlled simulations, LLMs frequently misinterpret or invalidate core psychological distress indicators such as suicidal ideation or trauma-related disclosures [3,4,5,6,7,8,9,10,11,12,13,14,15,16,17,18,19,20,21,22,23,24,25,26,27,28,29,30,31,32,33,34,35,36,37,38,39,40,41,42,43,44,45,46,47]. Furthermore, hallucinations, presented as plausible but false statements, are a serious risk in these contexts, where misinformation can have life or death consequences. This highlights the need for caution when relying on LLMs in clinical settings, where accurate interpretation of clinical signals is crucial.

### 10.2. Cultural Incompetence

The global deployment of AI tools often lacks cultural sensitivity, leading to the miscategorization of expressions of distress rooted in indigenous or spiritual traditions. For instance, Western-trained models may categorize such expressions as psychosis or delusion, undermining trust, reducing engagement, and perpetuating epistemic violence. This situation occurs when local knowledge is dismissed or pathologized, emphasizing the importance of culturally informed AI systems that can accurately understand and respond to diverse cultural contexts.

### 10.3. Ethical Deception and Emotional Illusions

The use of AI tools in therapy can create a false sense of emotional connection, leading users to over-disclose or over-trust systems incapable of safeguarding their well-being. Research suggests that many users cannot differentiate between AI-generated empathy and genuine human concern, creating a false therapeutic bond [17]. These research findings highlight the need for transparency and clear communication about the limitations of AI tools, ensuring that users understand the nature of the support they are receiving. From a cyberpsychology lens, individuals’ tendencies to anthropomorphize digital agents can intensify emotional attachment to AI, increasing the possibility of misplaced trust or dependency. Recent studies report that some chatbots endorsed harmful proposals in 19 out of the 60 (32%) opportunities to do so. Of the 10 chatbots, 4 endorsed half or more of the ideas proposed to them, and none of the bots managed to oppose them all, which supports careful oversight and clear communication of limits [8]. Moreover, the immersive and persistent nature of online interactions may deepen these emotional illusions, shaping user expectations and behaviors in ways distinct from traditional therapeutic relationships.

### 10.4. Privacy and Consent

The use of AI tools in therapy also raises concerns about privacy and consent. Without clear user education and explicit opt-in consent mechanisms, AI tools can violate foundational ethical principles such as autonomy and confidentiality. For example, AI chatbots often collect and store personal health information without offering users transparency or control, emphasizing the need for robust consent mechanisms and transparent data handling practices.

### 10.5. Legal and Accountability Gaps

The legal status of LLMs remains unclear, creating a paradox where harm is possible but redress is impossible. While disclaimers are used to avoid liability, users often interpret AI responses as medical advice, highlighting a clear breach of the right to recourse. This dynamic underscores the need for clear regulations and accountability mechanisms, ensuring that users have access to redress in cases where harm occurs.

### 10.6. Policy Implications

The rapid deployment of Large Language Models (LLMs) in therapeutic contexts has outpaced the development of coherent and enforceable policy frameworks, leaving significant ethical and clinical vulnerabilities unaddressed. AI regulation is beginning to emerge, such as the European Union’s AI Act and the United States Food and Drug Administration’s (FDA) digital health initiatives, but these efforts seem to lack specificity concerning therapeutic AI applications [23]. Recent updates to the EU AI Act classify health chatbots as high-risk systems, which mandates requirements for transparency, human oversight, data governance, and post-market monitoring. This framework directly applies to large language model–based mental health tools, placing them alongside other high-risk AI systems in healthcare. One core policy issue is the classification ambiguity of LLM-based tools. For example, are they medical devices, decision-support systems, or educational aids? This lack of clarity impedes the application of regulatory standards typically applied to therapeutic tools. However, FDA guidance addresses digital therapeutics and software as a medical device, particularly when clinical outcomes and safety claims are made. However, most wellness-oriented or experimental chatbots fall outside of this formal regulatory pathway, creating ambiguity about accountability and standards of evidence. The absence of a defined regulatory taxonomy enables developers to circumvent clinical evaluations by classifying their products as wellness or productivity applications, despite clear therapeutic intentions driving these products and user interpretations [12].

Furthermore, the cross-border deployment of LLMs presents serious jurisdictional and legal enforcement challenges. AI therapy tools hosted in one country are often used globally, yet no multilateral governance frameworks currently exist to ensure compliance with local ethical norms and clinical standards. Although the World Health Organization (WHO) has issued advisory guidelines on digital health ethics, they lack enforceability and depend on voluntary adoption [11]. Another concern is data governance and monetization. Many AI platforms rely on user interaction data to refine their models, yet few provide transparency regarding data storage, third-party access, or secondary use. The lack of transparency creates significant privacy vulnerabilities, especially under regimes such as the General Data Protection Regulation (GDPR) in the EU and the Health Insurance Portability and Accountability Act (HIPAA) in the U.S. While some national health agencies have proposed opt-in consent systems, compliance remains patchy and unstandardized [7].

Unlike pharmaceuticals, LLMs used in therapy are not subject to standardized trials, safety reporting, or post-deployment evaluation, and algorithmic certification is absent. A regulatory vacuum seems to persist, despite growing evidence that these systems can produce real harm in clinical and quasi-clinical environments [5], and this lapse in oversight and accountability raises serious concerns about patient safety and public trust. While oversight mechanisms do exist, they remain fragmented, unevenly enforced, and insufficiently tailored to adolescent mental health contexts. In practice, this means that while frameworks are in place, critical gaps persist in implementation and scope, leaving vulnerable populations under protected. Therefore, without urgent improvements in policy development to match the speed at which LLM Apps are deployed, these gaps will continue to compromise patient safety and public trust.

A harmonized policy architecture must emerge that treats therapeutic LLMs as high-risk interventions subject to licensing, review, and jurisdictional accountability. For policies to be effective, they must be part of a comprehensive and coordinated approach to regulating AI-based therapy, one that prioritizes patient safety, transparency, and accountability. By establishing clear guidelines and standards for the development, deployment, and evaluation of therapeutic LLMs, policymakers can help ensure that these technologies are used in ways that promote the well-being and safety of individuals, particularly vulnerable populations such as minors.

We align our recommendations with current clinical AI ethics and reporting guidance, including the American Psychological Association (APA) Ethical Principles of Psychologists and Code of Conduct, the Belmont Report’s principles of beneficence, nonmaleficence, and justice, WHO’s recommendations on digital interventions for health system strengthening, and emerging frameworks such as the National Institute for Health and Care Excellence (NICE) Evidence standards framework for digital health technologies. We call for pre-deployment risk assessment, ongoing audit, and public reporting of prompts and responses in clinical studies, which recent reviews and reporting tools propose for chatbot advice in care settings [48,49].

### 10.7. Age-Specific Regulations

The increasing use of Artificial Intelligence (AI) in mental health care has raised concerns about the regulation of AI applications, particularly in the context of minors. While broader AI regulations, such as the EU’s AI Act and the FDA’s digital health frameworks, are emerging, they often fail to address critical issues specific to pediatric and adolescent populations in therapeutic AI applications. One of the primary concerns is that adolescents occupy a unique legal space, being neither fully autonomous nor entirely dependent on adults.

However, current AI governance frameworks are predominantly designed with adult users in mind, leaving a regulatory gap for minors. The absence of age-specific regulatory mechanisms allows Large Language Model (LLM)-based tools to be accessed by minors without proper oversight. Many AI mental health applications include disclaimers stating that they are “not intended for clinical use.” However, these caveats are often not age-gated, nor are they enforced.

Research has shown that minors frequently use AI tools like Replika, Woebot, and Wysa for therapeutic purposes, often without parental knowledge or clinician awareness [7]. To enhance conceptual clarity, we distinguish four categories of AI tools relevant to mental health. Rule-based chatbots such as Woebot and Wysa deliver scripted cognitive behavioral prompts and do not qualify as large language models. Large language model chatbots such as ChatGPT and Claude generate free text and may be adapted for mental health purposes, but they lack the safeguards of clinically validated systems. Clinical decision support tools such as Babylon’s triage system are designed to assist clinicians rather than act as therapists. Companion agents such as Replika aim to provide mood support or social presence but are not clinical tools unless formally validated and regulated. It is also important to distinguish between research prototypes tested under controlled conditions and commercial applications available directly to consumers, as regulatory expectations, safety evidence, and real-world risks differ substantially for each AI tool. The lack of regulation and oversight raises concerns about the potential risks and benefits of AI use among minors. On one hand, AI-powered tools may provide accessible and engaging support for young people struggling with mental health issues.

On the other hand, the absence of proper guidance and monitoring may lead to potential harm, such as unsupervised use of AI tools that may not be suitable for minors, a lack of transparency in AI decision-making processes, and a potential for biased or inaccurate advice. To address these concerns, researchers and policymakers are exploring ways to develop more effective regulatory frameworks that consider the unique needs and vulnerabilities of minors. Some potential solutions include age-specific guidelines, enhanced transparency and explainability, and clinical validation and oversight. These measures aim to ensure that AI-powered tools are designed and used in ways that promote the well-being and safety of minors.

Furthermore, the issue of AI-based therapy involving minors is further complicated by the lack of legislative clarity around informed consent. In many jurisdictions, existing consent frameworks are tailored for human-to-human therapy and do not specify who bears responsibility for harm in AI interactions, especially those with children. Without legislative updates, minors may be exposed to therapeutic illusions that encourage over-disclosure and false expectations of confidentiality [12]. This ambiguity can lead to a lack of transparency and accountability, potentially putting minors at risk. In addition, the design architecture of Large Language Models (LLMs) rarely incorporates adolescent developmental psychology. Adolescent users express distress differently from adults via slang, metaphor, humor, or indirect cues. LLMs trained primarily on adult speech patterns or general internet corpora are prone to misunderstanding or trivializing these expressions, and these outcomes contribute to clinical misalignment and can exacerbate already fragile mental health conditions [11]. The mismatch between LLM training data and adolescent communication styles can lead to ineffective or even counterproductive therapy. Given these concerns, it is clear that age-sensitive regulation is not simply a supplementary measure but an essential safeguard in digital mental health governance. Policymakers must begin treating therapeutic AI interactions with minors as a high-risk clinical domain demanding specialized protections. This requires a proactive and nuanced approach to regulating AI-based therapy, one that takes into account the unique needs and vulnerabilities of minors. By prioritizing age-sensitive regulation, policymakers can help ensure that AI-based therapy is safe, effective, and beneficial for young people. Effective regulation will require a multidisciplinary approach, incorporating expertise from fields such as child development, psychology, and healthcare. It will also necessitate ongoing evaluation and monitoring of AI-based therapy, as well as collaboration between policymakers, researchers, and industry stakeholders. Ultimately, the goal of age-sensitive regulation is to create a safe and supportive environment for minors to access AI-based therapy while minimizing the risks and maximizing the benefits of these innovative technologies.

Developmental psychology highlights that adolescents, navigating Erikson’s stage of identity versus role confusion, are especially prone to anthropomorphizing and over-trusting relational agents, which heightens their susceptibility to therapeutic deception. Unlike adults, who typically draw on more stable self-concepts and broader social experience when interpreting ambiguous or metaphorical language, adolescents may misread chatbot responses as genuine empathy or authority. This contrast underscores the importance of embedding human backup and escalation protocols whenever LLMs are deployed with youth [50,51].

## 11. Institutional Risks

The integration of LLMs into mental health ecosystems is not only a technical or clinical matter but also an institutional liability issue, particularly in school systems, healthcare networks, and digital platform providers. Institutions that adopt or permit the use of AI-based mental health tools bear varying degrees of ethical, legal, and reputational risk. These risks are magnified when such tools are deployed in environments involving adolescents, a population that attracts heightened ethical scrutiny and statutory protection.

### 11.1. School Systems and Educational Institutions

A growing number of schools and universities are exploring the use of AI chatbots to augment overstretched mental health services. While such tools may appear to reduce staff burden and improve service availability, they can inadvertently expose schools to significant risks. For instance, in the absence of real-time human supervision, AI responses to sensitive disclosures such as abuse and suicidal ideation may be clinically inappropriate, morally negligent, or legally actionable. In jurisdictions with mandatory reporting laws for child endangerment, schools may face legal consequences if AI systems fail to flag at-risk youth. Further, deploying these tools without obtaining appropriate parental consent or institutional review board (IRB) oversight could violate student privacy regulations such as the US FERPA or the EU GDPR laws, particularly when student data is shared with third-party AI vendors.

### 11.2. Healthcare Institutions

Hospitals and clinics integrating LLM tools as pre-screening agents, telehealth assistants, or therapy augmenters must contend with regulatory ambiguity. If these tools are deployed without clear scope-of-practice definitions and without clinical protocols for escalation or intervention, the institutions risk malpractice exposure. A 2024 health-tech audit revealed that less than 20% of AI-integrated mental health deployments had formal risk management plans addressing adolescent use [52]. There is also the problem of interoperability, where AI systems often operate in silos, disconnected from electronic health records (EHRs), thereby fragmenting care and hindering accurate documentation. This fragmentation can undermine care continuity and lead to adverse outcomes if AI-generated responses contradict clinician decisions or delay appropriate human intervention. Model behavior is also not uniform; although recent advancements show improvements in psychiatric knowledge across newer models, wide variance still exists across products, including gaps in method reporting that limit direct comparison [10]. This paper reflects these differences in all claims of risk and benefit.

### 11.3. Technology Providers and Platform Hosts

Commercial developers of mental health chatbots increasingly operate in the space between wellness and clinical care, intentionally marketing their products to institutions while disclaiming therapeutic responsibility. This legal gray area, wherein companies claim not to provide therapy while simulating therapeutic interaction, has prompted calls for stricter accountability. Platform providers like app stores or web hosts may also face reputational damage if youth users are harmed while using unvetted AI tools. Without a certification standard or liability framework, it is unclear who should be held accountable in adverse events. Is it the developer, the institution, or the user? This institutional ambiguity invites both under-regulation and litigation, and also raises ethical questions about delegated responsibility, such as whether institutions are ethically justified in outsourcing mental health support to algorithms.

### 11.4. Research and Ethical Oversight Boards

Academic institutions and research teams developing LLMs for mental health applications have their own share of institutional risks. These include the potential for reputational harm if their tools are misused, for instance, data breaches during model training, and ethical violations in adolescent trials. Moreover, many ethics review boards lack the technical literacy to adequately assess the AI-specific risks, especially in adolescent populations who cannot fully consent or understand algorithmic limitations. To mitigate institutional risks, institutions should implement AI risk audits before deploying LLM-based tools, integrate adolescent use protocols into organizational policies, and conduct data governance and third-party compliance checks. IRBs must receive AI-specific training, particularly on child and adolescent safety. By proactively identifying and managing institutional vulnerabilities, organizations can reduce harm, strengthen trust, and model responsible AI governance.

## 12. Recommendations

To ensure the ethical, equitable, and safe integration of Large Language Models (LLMs) into mental health therapy, this paper advances five core recommendations as follows.

### 12.1. Mandated Clinical Oversight, Algorithmic Transparency and Third-Party Auditing, and Regulatory Certification for Therapeutic AI

LLMs should not operate autonomously in therapeutic contexts. Every AI-based interaction concerning mental health must be overseen or reviewed by a licensed mental health professional. This requirement should be encoded into national and regional digital health regulations. AI developers must disclose training datasets, algorithmic limitations, and risk thresholds. Independent third-party audits should assess models against benchmarks for bias, safety, and therapeutic coherence. A standardized certification mechanism must be introduced for LLMs marketed for therapeutic use, and these certifications, akin to drug licensing, should require initial validation, periodic renewal, and withdrawal in the event of adverse ethical outcomes.

### 12.2. Ethical Co-Design with Diverse Stakeholders, Global Governance and Interoperability Standards

AI systems must be co-developed with input from clinicians, ethicists, patients, and cultural experts. A stakeholder engagement co-development process helps address epistemic bias and promote context-sensitive systems. A global regulatory consortium, possibly under the WHO or a multilateral agency such as the OECD, should oversee AI therapy tools. Having a diverse team of experts would promote shared ethical standards and enable cross-jurisdictional enforcement. Any policy, regulatory, or technological discussion surrounding LLM use in therapy must include adolescent-specific safeguards. These include clinician oversight of all AI-adolescent interactions, training LLMs on adolescent-relevant communication styles and expressions, stronger consent mechanisms, including parental and youth assent, and clear boundaries in AI role definition. Addressing adolescent mental health needs is a central ethical obligation when deploying LLMs in global therapeutic landscapes. A failure to do so risks not only poor outcomes but also a loss of trust in future mental health innovations.

### 12.3. Adolescent-Specific Clinical Design—Age Verification and Use Restrictions

**Figure 2:**
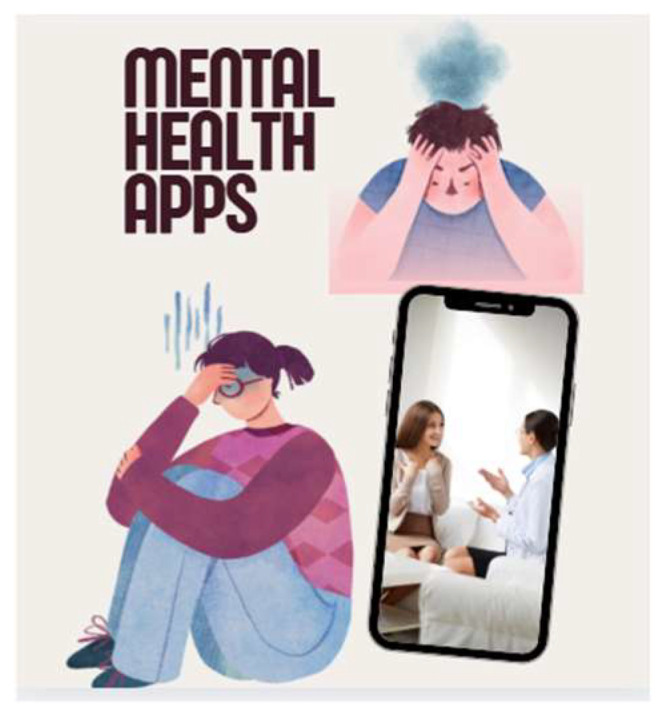
Graphical Illustration of Mental Health App Usage.

AI mental health tools must include robust age verification systems to prevent unsupervised use by individuals under 18. LLMs must be trained in developmentally appropriate language, emotional expression patterns, and common psychosocial concerns of adolescents, to prevent negative outcomes in users’ experience. Figure 2 provides a graphical illustration of how mental apps are used.

### 12.4. Enhanced Consent Protocols for Minors, Clinician Co-Presence, and Supervision Are Essential

The AI industry must adopt tiered consent protocols for adolescents, where interactions with LLMs are monitored and governed by dual consent, combining youth assent and guardian consent. Licensed child and adolescent therapists must oversee interactions either in real-time or asynchronously.

Data Sensitivity and Youth Privacy, Adolescent-Focused AI Ethics Charter should be implemented. Data governance policies must explicitly address adolescent data sensitivity. An international charter or framework, developed by bodies like the WHO or UNICEF, should articulate ethical principles for adolescent engagement in AI mental health tools.

## 13. Social Implications

The deployment of AI tools in mental health therapy has profound social implications, particularly in how emotional relationships, privacy, and trust are understood and managed within digitally mediated environments. As the paper highlights, adolescents and other vulnerable users often anthropomorphize chatbots and large language models (LLMs), forming perceived emotional bonds with systems that simulate, rather than embody, empathy. This perceived emotional intimacy can create a false sense of security, leading users to disclose sensitive information under the illusion of confidentiality or relational safety. The erosion of clear boundaries between humans and machines disrupts traditional social expectations about care, agency, and responsibility, particularly for younger users who may not yet have fully developed cognitive or emotional filters to navigate these blurred dynamics.

The normalization of therapeutic interactions with LLMs could also recalibrate broader cultural understandings of what constitutes authentic communication, care, and human connection. As AI becomes more embedded in mental health support systems, societies may begin to shift their expectations of relational reciprocity, potentially privileging speed and availability over depth and presence. These risks deepening social atomization, where emotionally hollow yet algorithmically responsive tools supplant genuine interpersonal support. The widespread, uncritical adoption of such systems without culturally responsive design or regulatory frameworks could accelerate a digital divide not only in terms of access, but in the quality and ethics of emotional care.

## 14. Implications on the Field of Therapy and Counseling

The proliferation of LLMs in mental health contexts introduces complex challenges to the therapeutic profession, including ethical boundary erosion, role confusion, and the commodification of care. As emphasized in the manuscript, AI systems are not governed by the same standards of accountability, licensure, or clinical oversight that human therapists must uphold. When users engage with AI as if it were a licensed counselor, they may expect the same safeguards, confidentiality, mandatory reporting, and ethical attunement, which LLMs cannot reliably provide. This misalignment not only threatens client safety but also jeopardizes the integrity of the therapeutic alliance, a cornerstone of effective counseling.

Moreover, the use of AI as a therapeutic proxy risks devaluing the nuanced competencies that trained therapists bring to the clinical encounter. Core skills such as empathic listening, interpretive flexibility, cultural humility, and attuned silence cannot be replicated through pattern-based language prediction alone. The manuscript underscores that AI’s frequent failures in handling complex disclosures, like suicidal ideation or trauma, reveal fundamental gaps in clinical reasoning and relational ethics. Suppose LLMs are marketed or adopted as sufficient substitutes for human therapists. In that case, the profession may face increasing pressure to redefine its scope not based on relational efficacy but on technological scalability and cost-efficiency, a shift that could erode the field’s evidence-based foundations.

## 15. Practical Implications

From a practical standpoint, the integration of LLMs into therapeutic settings presents immediate concerns around training, deployment, and oversight that must be addressed to avoid harm. As the paper notes, AI-generated responses can appear linguistically competent while remaining clinically inappropriate, such as offering trivial reassurances to suicidal users or misunderstanding idiomatic expressions of distress. In practical terms, this creates a false sense of security for both institutions deploying the tools and the individuals relying on them. Without robust systems of human moderation, crisis triage, and interpretive oversight, these tools may not only fail to help but actively harm users by delaying or misdirecting necessary care.

Additionally, there are logistical challenges in aligning AI tools with the varying regulatory and cultural landscapes across jurisdictions. For instance, an LLM trained on English-language forums may struggle with culturally specific idioms or therapeutic metaphors used in non-Western contexts, leading to inappropriate or offensive outputs. Practically, this calls for localized training data, multilingual design, and adaptive feedback loops to ensure relevance and safety. Institutions that fail to address these practical barriers risk not only reputational damage but also liability for foreseeable harm. The manuscript strongly suggests that until these practical guardrails are in place, widespread deployment of AI in mental health settings remains premature and ethically precarious.

## 16. Implications on Public Health

At the level of public health, the unchecked deployment of LLMs in mental health therapy could exacerbate existing disparities while introducing new vectors of systemic risk. As the manuscript outlines, LLMs are increasingly positioned as scalable solutions to therapist shortages and access gaps, particularly in low-resource settings. However, if these tools are deployed without proper safeguards, cultural calibration, and human oversight, they may deliver substandard care that reinforces marginalization rather than alleviates it. A chatbot misinterpreting an indigenous adolescent’s spiritual idiom as a psychotic symptom is not just a clinical failure; it is a public health injustice rooted in epistemic bias.

Worse still, if AI-driven systems fail to appropriately respond to acute risk, such as expressions of suicidality or abuse, the consequences can be fatal on a population scale. Public health systems depend on accurate, timely, and culturally attuned information to detect and respond to crises. AI tools that hallucinate, misdiagnose, or trivialize distress can erode the reliability of early intervention pipelines. The paper makes clear that these risks are not hypothetical; they have already been observed in case studies involving LLM deployments. Without regulatory interventions and interdisciplinary collaboration, public health systems may find themselves overwhelmed by harms introduced by technologies originally intended to extend care.

## 17. Future Research Directions

As the therapeutic applications of LLMs proliferate, future research must critically interrogate their long-term efficacy, equity, and safety.

### 17.1. Hybrid Human–AI Systems and Culturally Adaptive AI

Research should focus on the design and evaluation of hybrid systems, where AI augments human clinical decision-making but never replaces it. Controlled longitudinal studies should compare patient outcomes, therapeutic alliance quality, and ethical safety across augmented versus autonomous systems. AI tools must be embedded with culturally calibrated mental health ontologies. Future research should develop frameworks for localizing therapeutic models to respect linguistic, historical, and spiritual dimensions of psychological distress. Participatory research methods, including community-led AI prototyping, are essential in this effort. Additional directions include legal informatics analysis to establish liability frameworks for AI harm, psycholinguistic studies on AI-generated empathy and therapeutic illusion, and algorithmic audits focusing on racial, gender, and neurodivergence biases. An actionable research agenda must guide not only technical innovation but also ethical reflection and social justice orientation.

### 17.2. Adolescent-Centric Approaches

When it comes to adolescents, specific considerations must be taken into account. All AI systems that engage with users aged 13–18 must operate under licensed clinical oversight. Engaged oversight should include human review of interactions involving severe symptom disclosures such as self-harm, suicidal ideation, and abuse. Systems should include escalation flags that trigger emergency responses tailored to adolescent legal rights and privacy protections. Training data must reflect adolescent-specific communication styles, including slang, indirect expression, and contextual cues. Adolescent users often use metaphor or irony to describe distress, which LLMs misinterpret. AI developers should collaborate with youth clinicians and adolescent users to create models attuned to teen affective speech patterns.

Furthermore, LLMs should disclose their non-human, non-clinician status at the start of every interaction with minors. Transparent disclosure prevents therapeutic deception and over-trust. Systems should also reiterate their limitations in responding to emergencies and clarify when human help is needed.

### 17.3. Dual Consent, Access Control, and Culturally Adapted Adolescent Versions

Adolescent engagement with AI mental health tools should involve dual-consent protocols combining user assent with optional parental involvement, as per regional health regulations. Developers should provide parental dashboards for monitoring usage without compromising user confidentiality, offering a balanced approach to youth autonomy and parental oversight. Adolescent populations are not monolithic; therefore, LLMs must be customized across cultural, linguistic, and socioeconomic contexts to reflect differing norms around mental health, emotional expression, and help-seeking.

### 17.4. Adolescent-Centered Evaluation Metrics

Evaluation frameworks must go beyond general usability and measure adolescent-specific outcomes: emotional safety, developmental appropriateness, and therapeutic clarity. These should be validated through co-design research involving youth participants and child mental health experts.

### 17.5. Lessons from Pilot Real-World Implementations of AI in Mental Health Care

The following mini cases are illustrative and are used to explain typical risks and safeguards. Where a peer-reviewed primary source exists, we cite it, and where such a source does not exist, we state that the example is illustrative.

*NHS-Babylon Pilot (United Kingdom)*—The integration of AI in mental health care has yielded varying results, as evident in several real-world implementations. One notable illustration is the NHS-Babylon pilot in the United Kingdom, which aimed to alleviate primary care referral waitlists by using an AI chatbot to evaluate users’ self-reported symptoms and recommend triage paths. However, the system frequently misclassified cases involving subtle emotional distress and psychosocial complexity, failing to interpret emotional nuance, sarcasm, or ambivalence accurately. The lack of psychiatric oversight during model design and deployment created epistemic blind spots that directly affected user outcomes [6].

*South Korean Clinical Decision Support Tool (CDST)*—Ref. [13] provided a more realistic example in the study of a group therapy augmentation tool deployed in South Korea, where therapy was supported by a generative AI-enabled tool alongside human-led group sessions. The AI tool did not replace therapists but served to enhance patient engagement: it delivered personalized prompts and supplementary materials, tracked attendance, and aided in retaining participants, which led to lower dropout rates and higher session attendance among users of the tool compared to controls. While the study did not involve automatic transcription of therapy sessions, affective tone analysis, or flagging of cognitive distortions for therapist review as part of the AI’s capabilities, the findings do underscore the benefits of augmentation over substitution. The human-led therapeutic alliance remained central; the AI served to support therapists by reducing some burden (such as preparing supportive content and follow-ups) rather than replacing clinical judgment. This model demonstrates that integrating AI as a supplement to human therapists can meaningfully improve engagement and outcomes without compromising therapeutic trust [13].

The importance of cultural competence and linguistic localization can be illustrated by a less successful example such as from a Latin American university, where an English-trained mental health chatbot was deployed to serve Spanish-speaking students without proper adaptation. The system would misinterpret idioms, fail to detect suicidal risk in metaphoric language, and provide inadequate coping strategies, resulting in a sharp backlash and eventual deactivation. Post-analysis attributes such failures to ethnocentric training data and a lack of human testing with local populations [12]. This example is presented as an internal hypothetical scenario to illustrate governance choices in low-resource academic clinics where peer-reviewed pilot reports are limited. Recent youth-focused evaluations of mental health chatbots highlight both promise and careful limits which inform this scenario design [14].

### 17.6. Key Takeaways and Implications

These three cases reveal the decisive role of context, cultural competence, and oversight in determining the ethical and clinical viability of LLMs in therapy. Systems that ignore these components risk harm, while systems that integrate them enhance care. The importance of careful design, deployment, and evaluation of AI systems in mental health care cannot be overstated, and these examples serve as valuable lessons for future implementations.

## 18. Conclusions

The proliferation of Large Language Models (LLMs) in mental health care reflects a convergence of technological enthusiasm, systemic need, and digital optimism. However, their deployment without rigorous ethical frameworks, clinical oversight, and cultural adaptation presents profound risks, ranging from diagnostic missteps to privacy violations and therapeutic deception. Grounded in the Ethics of Care and the Augmented Intelligence paradigm, this paper argues that LLMs must be understood as supportive tools, not autonomous therapists, and their value lies in enhancing clinician efficiency, improving access in low-resource settings, and enabling proactive screening. To realize these benefits, enforceable regulatory standards, algorithmic transparency, culturally calibrated design, and a commitment to non-maleficence are necessary. This paper critically examines the empirical limitations, ethical concerns, and regulatory gaps surrounding AI-assisted mental health care and proposes a structured policy and LTGM implementation framework that prioritizes risk mitigation, algorithmic transparency, and clinician-guided oversight. The framework emphasizes hybrid systems that augment, rather than replace, human therapists, cultural adaptation in low-resource settings, and the development of globally harmonized governance models. Ultimately, the future of AI in mental health therapy depends on ethical integration, contextual sensitivity, and an unwavering commitment to human dignity and safety, rather than technological superiority. If integrated responsibly, LLMs may find a dignified place in the global mental health ecosystem as allies, not impostors, thereby transforming the tools of healing into valuable assets rather than instruments of harm.

## Data Availability

No new data were created or analyzed in this study. Data sharing is not applicable to this article.

## Supplementary Materials

The following supporting information can be downloaded at: https://www.mdpi.com/article/10.3390/healthcare13212721/s1, The supplementary materials contains details of the following theories which were integrated into the paper, Constructive Dialogue Theory [24,25,26], Normative Moderation Theory [26,27], Psychosocial Development Theory [28], Relational-Cultural Theory (RCT) [29,30], Relational Dialectics Theory [31,32], Expectancy Violations Theory [33,34], Social Constructionism [35,36], Goffman’s Dramaturgical Theory [37,38], Social Cognitive Theory [40], Actor-Network Theory [41], and the Ecological Systems Theory [42].

## References

[1] World Health Organization (2022). *Mental Disorders* [Fact Sheet]. https://www.who.int/news-room/fact-sheets/detail/mental-disorders.

[2] Ohu F.C., Jones L.A. (2025). The Intersection of Cyberwarfare, Social Media, and Adolescent Self-Esteem: A Forensic Cyberpsychology Analysis. Proceedings of the 20th International Conference on Cyber Warfare and Security (ICCWS 2025).

[3] Prichett L.M., Yolken R.H., Severance E.G., Carmichael D., Zeng Y., Lu Y., Young A.S., Kumra T. (2024). COVID-19 and Youth Mental Health Disparities: Intersectional Trends in Depression, Anxiety, and Suicide Risk-Related Diagnoses. Acad. Pediatr..

[4] Racine N., McArthur B.A., Cooke J.E., Eirich R., Zhu J., Madigan S. (2021). Global Prevalence of Depressive and Anxiety Symptoms in Children and Adolescents During COVID-19: A Meta-Analysis. JAMA Pediatr..

[5] Schnepper R., Roemmel N., Schaefert R., Lambrecht-Walzinger L., Meinlschmidt G. (2025). Exploring Biases of Large Language Models in the Field of Mental Health: Comparative Questionnaire Study of the Effect of Gender and Sexual Orientation in Anorexia Nervosa and Bulimia Nervosa Case Vignettes. JMIR Ment. Health.

[6] Iacobucci G. (2020). Babylon’s chatbot: GPs express concerns about safety. BMJ.

[7] Rahsepar Meadi M., Sillekens T., Metselaar S., van Balkom A., Bernstein J., Batelaan N. (2025). Exploring the Ethical Challenges of Conversational AI in Mental Health Care: Scoping Review. JMIR Ment. Health.

[8] Clark A. (2025). The ability of AI therapy bots to set limits with distressed adolescents: Simulation-based comparison study. JMIR Ment. Health.

[9] Kim J., Vajravelu B.N. (2025). Assessing the current limitations of large language models in advancing health care education. JMIR Form. Res..

[10] Huo B., Boyle A., Marfo N., Tangamornsuksan W., Steen J.P., McKechnie T., Lee Y., Mayol J., Antoniou S.A., Thirunavukarasu A.J. (2025). Large language models for chatbot health advice studies: A systematic review. JAMA Netw. Open.

[11] Vertsberger D., Naor N., Winsberg M. (2022). Adolescents’ well-being while using a mobile artificial intelligence-powered acceptance commitment therapy tool: Evidence from a longitudinal study. JMIR AI.

[12] Bak M.A.R., Schut M.C., Ploem M.C. (2025). Embedding Ethical and Legal Principles in AI-Driven Clinical Practice: Two Use Cases in Laboratory Diagnostics. J. Responsib. Technol..

[13] Habicht J., Dina L., McFadyen J., Stylianou M., Harper R., Hauser T., Rollwage M. (2025). Generative AI–enabled therapy support tool for improved clinical outcomes and patient engagement in group therapy: Real-world observational study. J. Med. Internet Res..

[14] Balan R., Dobrean A., Poetar C.R. (2024). Use of automated conversational agents in improving young population mental health: A scoping review. npj Digit. Med..

[15] Donia J., Shaw J.A. (2021). Co-design and Ethical Artificial Intelligence for Health: An Agenda for Critical Research and Practice. Big Data Soc..

[16] Villegas-Galaviz C., Martin K. (2024). Moral Distance, AI, and the Ethics of Care. AI Soc..

[17] Joseph A.P., Babu A. (2024). Transference and countertransference with artificial intelligence chatbots in mental health. Front. Psychiatry.

[18] Karkosz S., Szymański R., Sanna K., Michałowski J. (2024). Effectiveness of a web-based and mobile therapy chatbot on anxiety and depressive symptoms in subclinical young adults: Randomized controlled trial. JMIR Form. Res..

[19] Hanss K., Sarma K., Glowinski A., Krystal A., Saunders R., Halls A., Gorrell S., Reilly E. (2025). Assessing the accuracy and reliability of large language models in psychiatry using standardized multiple-choice questions: Cross-sectional study. J. Med. Internet Res..

[20] Venerito V., Gupta L., Mileto S., Iannone F., Bilgin E. (2025). Ethical Challenges and Regulatory Pathways for Artificial Intelligence in Rheumatology. Rheumatol. Adv. Pract..

[21] Weiner E.B., Dankwa-Mullan I., Nelson W.A., Hassanpour S. (2025). Ethical Challenges and Evolving Strategies in the Integration of Artificial Intelligence into Clinical Practice. PLoS Digit. Health.

[22] World Health Organization (WHO) (2025). Improving the Mental and Brain Health of Children and Adolescents. https://www.who.int/activities/improving-the-mental-and-brain-health-of-children-and-adolescents.

[23] Sangaraju V.V. (2025). AI and Data Privacy in Healthcare: Compliance with HIPAA, GDPR, and Emerging Regulations. Int. J. Emerg. Trends Comput. Sci. Inf. Technol..

[24] Keyan D., Hall J., Jordan S., Watts S., Au T., Dawson K.S., Abu Sway R., Crawford J., Sorsdahl K., Luitel N.P. (2025). Development of a World Health Organization digital mental health intervention for adolescents in low- and mid-dle-income countries: A human-centered design approach. Front. Digit. Health.

[25] Isaacs W.N. (2001). Toward an Action Theory of Dialogue. Int. J. Public. Adm..

[26] Pu I., Ravi P., Dinh L.D., Joe C., Ogoe C., Li Z., Breazeal C., Ostrowski A.K. “How Can We Learn and Use AI at the Same Time?”: Participatory Design of GenAI with High School Students. Proceedings of the 24th Interaction Design and Children.

[27] Chen J., Xia S., Lin T. (2024). A Framework of Moderators in Social Norm-Based Message Persuasiveness Based on a Systematic Review. Hum. Commun. Res..

[28] Horstkötter D., Kanne M., Karbouniaris S., Lazrak N., Bulgheroni M., Sheltawy E., Giani L., La Gamba M., Pujadas E.R., Camacho M. (2025). Decision-Making on an AI-Supported Youth Mental Health App: A Multilogue Among Ethicists, Social Scientists, AI-Researchers, Biomedical Engineers, Young Experiential Experts, and Psychiatrists. J. Responsible Technol..

[29] Murad R.J. (2024). Investigating the Impact of Digital Technology on Adolescent Identity Formation on Selected Students in SAIS: A Psychological Approach. Int. J. Innov. Sci. Res. Technol..

[30] Evans K., O’Donnell K. (2025). Relational Cultural Theory and Intervention Approaches with Adolescent Girls: An Integrative Review. Affilia.

[31] Koffmann A. (2023). Early Distress Score Instability Predicts Outcome in Brief Psychotherapy. J. Couns. Psychol..

[32] Scharp K.M., Thomas L.J., Baxter L.A., Braithwaite D.O. (2021). Relational Dialectics Theory: A Dialogic Theory of Meaning-Making. Engaging Theories in Interpersonal Communication.

[33] Zou W., Yang X., Liu Z. (2025). Scientific Tool or Torture Device? A Relational Dialectics Theory Approach to the Speculum’s Symbolism in China. J. Soc. Pers. Relatsh..

[34] Chen Y., Li X. (2024). Expectancy Violations and Discontinuance Behavior in Live-Streaming Commerce: Exploring Human Interactions with Virtual Streamers. Behav. Sci..

[35] Rheu M., Dai Y., Meng J., Peng W. (2024). When a Chatbot Disappoints You: Expectancy Violation in Human-Chatbot Interaction in a Social Support Context. Commun. Res..

[36] Issar S. (2024). The Social Construction of Algorithms in Everyday Life: Examining TikTok Users’ Understanding of the Platform’s Algorithm. Int. J. Hum. Comput. Interact..

[37] Paganelli M. (2024). Identity Construction and Representation in the Digital Age: A Gen Z Social Media Perspective. Preprint.

[38] Steele Gray C., Shaw J., Baker G.R., Kuluski K., Wodchis W.P. (2024). The Integrated Care World is a Stage: Applying Goffman’s Theory of Dramaturgy to the Activities of Integrated Care. Int. J. Integr. Care.

[39] Jin Y., Tian Y., Wu H. (2022). The presentation of self on online social network platforms from the perspective of dramaturgical theory: Taking WeChat Moments and anonymous question platform “Tape” as examples. Proceedings of the 2021 International Conference on Social Development and Media Communication (SDMC 2021).

[40] Kaplan D. (2024). Performing Identity or Performing Relationships? Rethinking Performance Theory in Social Media Studies. Cult. Sociol..

[41] Amsari D., Wahyuni E., Fadhilaturrahmi F. (2024). The Social Learning Theory Albert Bandura for Elementary School Students. J. Basicedu.

[42] Ryan T., Ryan N., Hynes B. (2024). The Integration of Human and Non-Human Actors to Advance Healthcare Delivery: Unpacking the Role of Actor-Network Theory, a Systematic Literature Review. BMC Health Serv. Res..

[43] Rose T., Lambert S., Liu C., Raghunathan R.S., Musci R.J., Sullivan A.D.W., Lyall K., Elliott A.J., McEvoy C.T., Frazier J.A. (2025). Socio-Ecological Domains and Adolescent Mental Health: An Application of the Dual-Factor Model. J. Res. Adolesc..

[44] Koskela-Huotari K. (2024). How to craft a compelling storyline for a conceptual paper. AMS Rev..

[45] Chiat J., Ong L., Ning Y., Liu M., Ma Y., Liang Z., Singh K., Chang R.T.’, Vogel S., Lim J.C.W. (2025). Regulatory Science Innovation for Generative AI and Large Language Models in Health and Medicine: A Global Call for Action. arXiv.

[46] Heston T.F. (2023). Safety of Large Language Models in Addressing Depression. Cureus.

[47] Li T., Yang S., Wu J., Wei J., Hu L., Li M., Wong D.F., Oltmanns J.R., Wang D. (2025). Can Large Language Models Identify Implicit Suicidal Ideation? An Empirical Evaluation. arXiv.

[48] Wang S., Cheng Y., Song A., Keedy S., Berman M., Feamster N. (2025). Can LLMs address mental health questions? A comparison with human therapists. arXiv.

[49] Hong M., Kang R., Yang J., Rhee S., Lee H., Kim Y., Lee K., Kim H., Lee Y., Youn T. (2024). Comprehensive symptom prediction in inpatients with acute psychiatric disorders using wearable-based deep learning models: Development and validation study. J. Med. Internet Res..

[50] Ko C., Kim S. (2024). Adolescent Female Users’ Avatar Creation in Social Virtual Worlds: Opportunities and Challenges. Behav. Sci..

[51] Ohu F.C., Jones L.A. AI-Driven Forensic Cyberpsychology Intervention Strategies for Social Media Platform and School Managers to Mitigate Cyber Fraud At-Risk Adolescents. Proceedings of the Scientia Moralitas Conference.

[52] Ojewale V., Steed R., Vecchione B., Birhane A., Raji I.D. Towards AI Accountability Infrastructure: Gaps and Opportunities in AI Audit Tooling. Proceedings of the Conference on Human Factors in Computing Systems, Association for Computing Machinery.

