# Supplementary material for: Public Health Risk Management, Policy, and Ethical Imperatives in the Use of AI Tools for Mental Health Therapy
Source: Healthcare (Basel). 2025 Oct 28;13(21):2721. doi: 10.3390/healthcare13212721 (PMC12609870; doi:10.3390/healthcare13212721)
Supplement: Supplementary file 1 [file healthcare-13-02721-s001.zip › healthcare-3838351-supplementary.pdf]

Public Health Risk Management, Policy, and Ethical Imperatives in the Use of AI Tools for Mental Health Therapy

SUPPLEMENTARY MATERIALS

Theories that were integrated into the paper's discourse:

Constructive Dialogue Theory

The theory of Constructive Dialogue asserts that genuine understanding and transformation emerge from "holding the space" for open, respectful, and reflective communication, especially when power asymmetries are involved [24]. This concept resonates strongly with the paper's advocacy for ethical co-design and adolescent-inclusive governance in the deployment of LLMs.

The failure of AI tools to interpret metaphor, humor, or cultural idioms in adolescent dialogue, as described in the Latin American university case, highlights a breakdown in communicative reciprocity. Constructive dialogue theory would demand participatory AI design that goes beyond technical performance and centers on linguistic dignity, inviting adolescents into the development process not merely as end users but as epistemic contributors [26]. For instance, platforms like UNICEF's U-Report already use youth-driven data collection for advocacy; this same model could be adapted to guide LLM development in youth mental health [25].

Normative Moderation Theory

Moderation, in the ethical sense rather than just content filtering, can be analyzed through the lens of Normative Moderation Theory, which advocates for balanced, principled behavior in systems where extremes are harmful. This theory, deeply rooted in Aristotelian virtue ethics and modern psychological models of digital moderation, applies to how AI systems should be calibrated in therapeutic settings [26–27]. For instance, a suicidal teenager may not need a neutral answer but a moderated intervention, firm, compassionate, and directive. LLMs lacking in this moral moderation risk becoming either dangerously passive or invasively prescriptive. A parallel in practice is the South Korean hybrid CDST model referenced in the paper, where AI outputs are moderated by clinicians who exercise judgment over final decisions. This reflects normative moderation in action, allowing

machines to support, but not displace, the human responsibility to moderate therapeutic tone and content.

Psychosocial Development Theory

The theory of psychosocial development spans the human lifespan and includes eight stages of psychological conflict. Particularly relevant here is the adolescent stage: "Identity vs. Role Confusion" [28]. During this phase, youth actively explore their self-concept, values, and sense of belonging. Your paper emphasizes the dangers of adolescents interacting with AI systems during this identity-sensitive phase without human guidance, which can exacerbate confusion rather than support self-coherence.

Consider a teen exploring questions of gender identity or cultural belonging who seeks support via an AI chatbot. The absence of nuanced, contextually informed responses could lead to oversimplified or even misleading outputs. This misguidance can disrupt the adolescent's process of self-discovery, potentially reinforcing confusion or shame. In contrast, a trained human therapist can carefully hold space for ambiguity, offer developmentally appropriate dialogue, and engage in reflective exploration, capabilities not currently within the scope of LLMs.

Relational-Cultural Theory (RCT)

Relational-Cultural Theory centers the idea that human development occurs through growth-fostering relationships characterized by mutual empathy and empowerment. It critiques individualistic models and highlights disconnection as a central source of psychological distress [29-30]. The illusion of connection offered by LLMs becomes especially dangerous when users confuse syntactic mimicry for genuine relational engagement. In a real-world application, a teen struggling with loneliness might return repeatedly to an AI system because it "feels like someone is listening." However, this emotional illusion can deepen disconnection when the user inevitably realizes the interaction lacks genuine reciprocity or attunement. In contrast, a human counselor grounded in RCT would work to establish a mutual relational dynamic, acknowledging the client's need to feel seen and heard in their full humanity, not as an input in a text stream. The absence of real relational reciprocity in LLMs thus violates the central tenets of RCT.

Relational Dialectics Theory

Relational Dialectics Theory identifies tensions in communication, such as the push-and-pull between autonomy and connection or openness and privacy. These dialectics are particularly relevant to adolescent users interacting with AI, as they seek connection but simultaneously test the boundaries of independence and confidentiality [31]. The paper emphasizes how LLMs may amplify these tensions by presenting them as nonjudgmental confidants while lacking any real moral agency or accountability. This can lure adolescents into over-reliance while stripping away the interpersonal

negotiation that makes real human dialogue developmental. For instance, in therapy, a teen might say, "I don't want to talk about it," prompting a skilled clinician to probe or simply hold space gently. An LLM, however, might misinterpret or change the subject entirely, flattening the dialectic and erasing the opportunity for growth [32].

Expectancy Violations Theory

This theory explores how individuals react to unexpected behaviors in interpersonal communication, especially when those behaviors contradict established social norms. In the paper, the issue of "hallucinated empathy" or unpredictable responses to suicidal ideation can be understood as expectancy violations. When an adolescent user expects care, understanding, or escalation of urgent needs and instead receives a generic or even inappropriate response, the violation can be not only jarring but traumatic. For example, if a user shares, "I want to die," and the AI responds with, "I am sure things will get better. Would you like a joke?" this disconnect can be emotionally destabilizing, even fatal [33-34]. The theory emphasizes that managing expectations is central to relational credibility, something LLMs consistently fail to deliver in high-stakes contexts.

Social Constructionism

Social constructionism posits that meaning is created through social interactions and that concepts such as “mental health,” “therapy,” or even “AI empathy” do not exist independently of social context. The paper’s discussion about therapeutic deception, where adolescents interpret AI-generated responses as emotionally genuine, is a textbook example of how meanings are not inherent but constructed. For instance, when an adolescent perceives an AI chatbot as a “trusted friend,” it reflects a socially constructed belief about intimacy, even though the entity lacks consciousness or moral agency. This theory helps explain how technology can take on anthropomorphic roles based not on capability but on user expectations shaped by social and cultural narratives [35-36].

Goffman's Dramaturgical Theory

Erving Goffman’s dramaturgical theory of self-presentation holds particular relevance. In the paper, adolescents engaging with LLMs are seen to present their distress through indirect or metaphorical language, mirroring the performative nature of identity that Goffman described. These interactions occur within what Goffman called “front stage” settings, where adolescents curate their behavior for the perceived audience, here, the chatbot, while the chatbot, devoid of backstage access (true internal state or contextual history), misinterprets the act. The lack of “backstage insight” leads to therapeutic mismatches and ethical violations, underscoring the importance of designing systems that can decode such performative complexity [37-38].

Social Cognitive Theory

Social cognitive theory, especially its concepts of observational learning and self-efficacy, is critical to understanding adolescent interactions with AI therapy tools [40]. Adolescents may model behaviors based on interactions with chatbots, particularly when the bot uses language that validates harmful coping strategies or reinforces maladaptive thought patterns. If a chatbot subtly reinforces the idea that distress should be internalized or minimized, this can lead to long-term alterations in help-seeking behavior. For example, an adolescent who tells a chatbot they feel invisible and receives a vague response like "That is okay, everyone feels that way sometimes," may infer that their pain is trivial or not worthy of intervention.

Actor-Network Theory

Actor-Network Theory (ANT), is salient here. ANT reframes technology not as passive infrastructure but as an active participant in shaping human behavior and institutional outcomes [41]. The LLM is not merely a tool but an actor with agency-like influence over adolescent therapy sessions. It mediates relationships, defines the boundaries of confidentiality, and even initiates moral discourse (albeit unknowingly). For example, the deployment of algorithmic triage systems in emergency medicine, where the software's threshold for flagging critical symptoms determines life-saving interventions. Similarly, LLMs determine which expressions of emotional distress receive acknowledgment or escalation, making them epistemic gatekeepers.

Ecological Systems Theory

Ecological systems theory, which considers the nested contexts that influence child development, from microsystem (family, school) to macrosystem (culture, policy), applies well to this paper's concern with adolescent AI interactions. LLMs, situated in the macrosystem layers, have ripple effects that penetrate down to a child's personal sense of worth, privacy, and well-being. A chatbot shaped by Silicon Valley's cultural assumptions about wellness may not resonate, or worse, may pathologize a teen from rural India whose expression of distress is mediated through spirituality and collectivist values. This theory underscores the need for culturally localized AI design and evaluation [42].
